# Supplementary material for: Stress landscape of folding brain serves as a map for axonal pathfinding
Source: Nat Commun. 2025 Jan 30;16:1187. doi: 10.1038/s41467-025-56362-3 (PMC11782574; doi:10.1038/s41467-025-56362-3)
Supplement: Supplementary file 1 — Supplemenraty Information [file 41467_2025_56362_MOESM1_ESM.pdf]

Supplementary Information

Stress Landscape of Folding Brain Serves as a Map for Axonal Pathfinding

Akbar Solhtalab[1], Ali H. Foroughi[1], Lana Pierotich[2], Mir Jalil Razavi[1]

[1]Department of Mechanical Engineering, State University of New York at Binghamton, Binghamton, NY, USA

[2]Division of Newborn Medicine, Boston Children's Hospital, Harvard Medical School, Boston, MA, USA

*Corresponding Author: mrazavi@binghamton.edu

ORCID of M.J. Razavi: https://orcid.org/0000-0002-8421-9688

ORCID of A. Solhtalab: https://orcid.org/0009-0006-4766-3727

Table S1. The timeline for the development of thalamocortical fiber tracts, extracted from Krsnik et al. [1]. GW: gestational week.

GWs	Process
10	Axon outgrowth starts
10-11.5	Axons form massive bundles that traverse the diencephalic-telencephalic boundary
11.5-13	Axons pass the periventricular area at the pallial-subpallial boundary and enter intermediate zone in radiating fashion.
14-16	Axons, aligned along the fibers from the basal forebrain, continue to grow for a short distance within the deep intermediate zone and enter the deep CP, parallel with SP expansion.
16-20	Axons interdigitate with callosal fibers, running shortly in the sagittal stratum and spreading through the deep SP ("waiting" phase).
21-24	Axons accumulate in the superficial SP below the somatosensory cortical area*
25-26	Axons penetrate the CP concomitantly with its initial lamination.
27-36	Reactivity of the CP exhibits an uneven pattern suggestive of vertical banding, showing a basic 6-layer pattern. Settlement stage.

* This occurs 2 weeks earlier than in the frontal and occipital cortices.

Table S2. Parameters and their values used in the simulations

Parameter	Cortical thickness (mm)	Axon growth rate (mm d^{-1})	Stiffness ratio of the cortex to the ECM (μ_c/μ_s)	Stiffness ratio of fibers to the ECM (μ_f/μ_s)	stress-dependent axon elongation rate a (mm Pa^{-1} d^{-1})	Standard deviation of the growth angle (Noise) (rad)
Values	1.5	0.6, 0.8, 1.0, 1.2	1, 2, 3, 4	1, 2, 4, 5	0.015	0.025 − 0.125

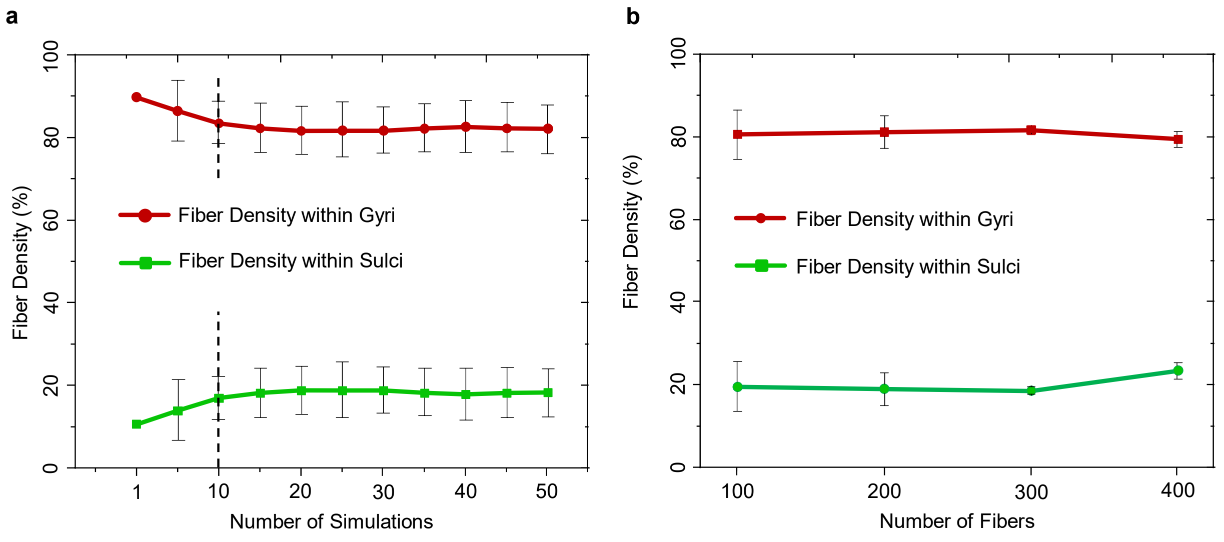

Fig. S1 Effect of simulation count and axon fiber bundle number on fiber density in gyri and sulci. **a** The impact of varying simulation counts on fiber density within gyri and sulci regions. Results demonstrate stability in fiber density beyond a threshold of 10 simulations. **b** Relationship between the number of fiber bundles and the resulting fiber density. The number of fibers doesn't significantly impact the fiber density within gyri and sulci. The used parameters in these figures are: $\mu_f/\mu_s = 2$, $\mu_c/\mu_s = 2$, $a = 0.015\ \mathrm{mm\ Pa^{-1}\ d^{-1}}$, $G^{axn} = 0.8\ \mathrm{mm\ d^{-1}}$, where μ_f, μ_s, and μ_c are the shear moduli of the fiber, ECM, and cortex, respectively, a is the stress-dependent elongation rate, and G^{axn} is the axon growth rate. Data are represented as mean values +/- SD (Standard Deviation). Source data are provided as a Source Data file.

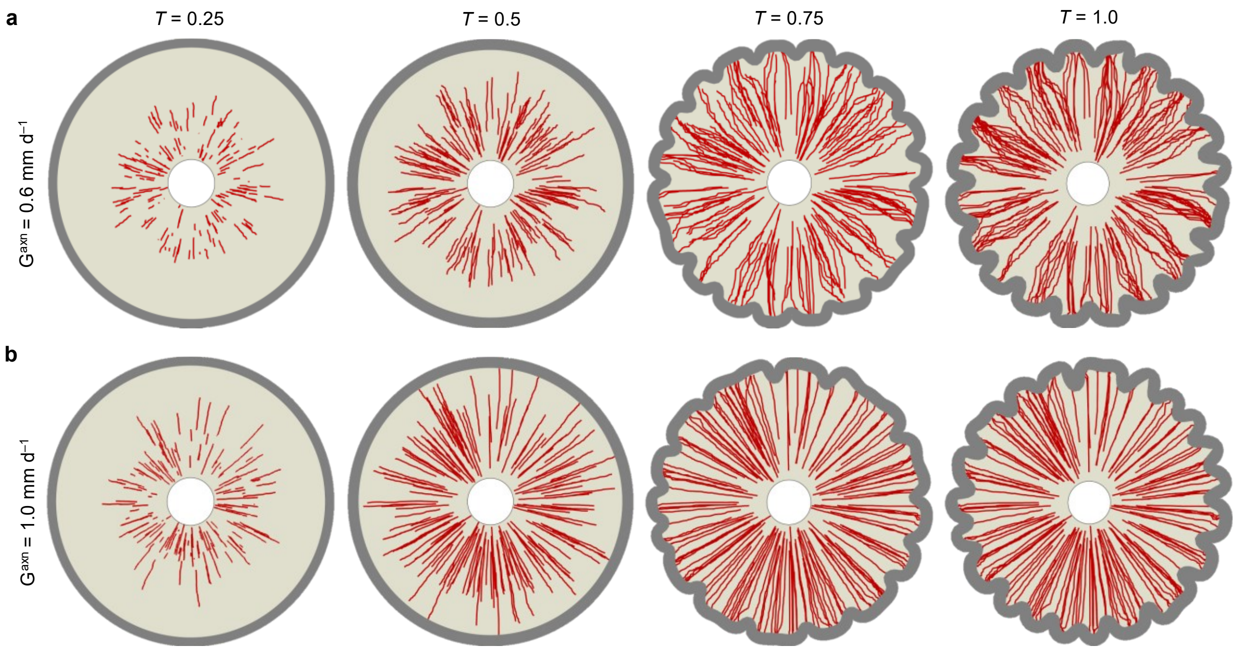

Fig. S2 Dynamic Growth and Pathfinding of Fibers in a Folding 2D Circular System at Different Growth Rates. Dynamic growth and pathfinding of fibers for two different axon growth rates, (a) $G^{\mathrm{axn}} = 0.6\,\mathrm{mm\,d^{-1}}$, and (b) $G^{\mathrm{axn}} = 1.0\,\mathrm{mm\,d^{-1}}$. As shown, the fibers grow and orient more toward the gyri than the sulci, consistent with the behavior observed in the rectangular models. $T = G^{\mathrm{ctx}}t$ is dimensionless simulation time that ranges from 0 to 1, where G^{ctx} and t are cortex growth rate and time, respectively.

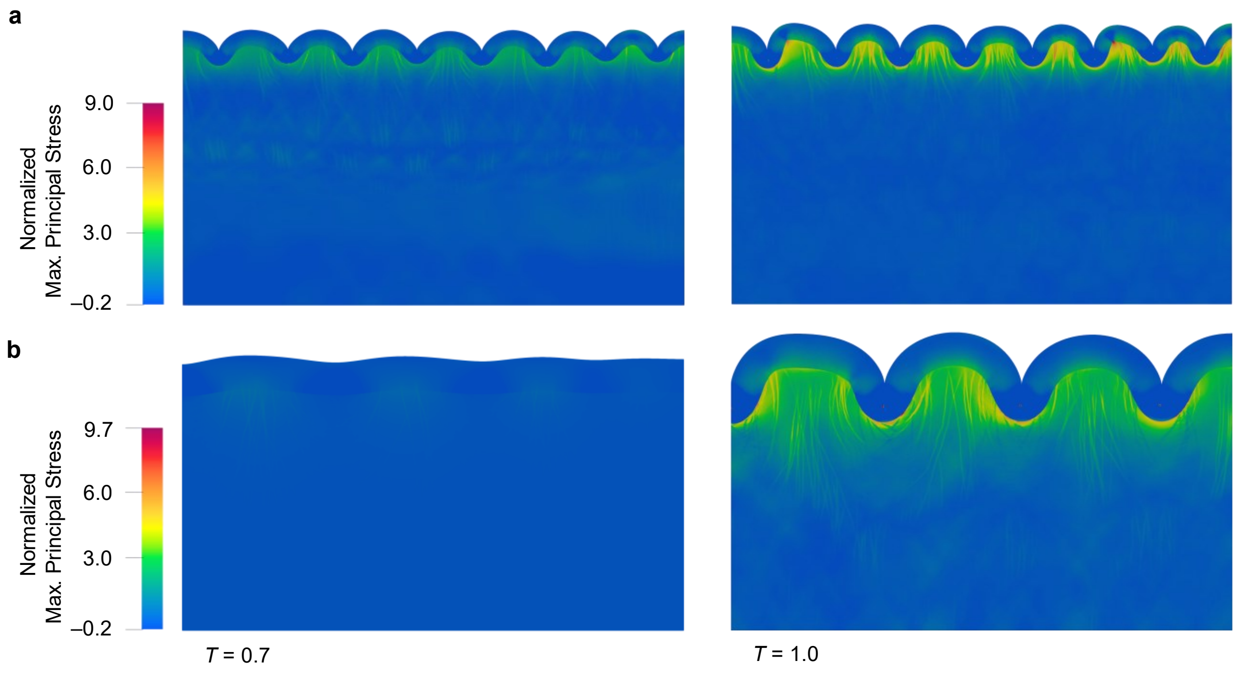

Fig. S3 Cortical folding patterns across varying cortical layer thicknesses. Cortical folding and normalized maximum principal stress map for an initial cortex layer thickness of **a** 1.5 mm, and **b** 3 mm. In both

scenarios, all other parameters remained constant: $G^{\mathrm{axn}} = 0.8\ \mathrm{mm\ d^{-1}}$, $\mu_c/\mu_s = 2$, $a = 0.015\ \mathrm{mm\ Pa^{-1}\ d^{-1}}$, where G^{axn} is the axon growth rate, μ_c and μ_s are the shear moduli of the ECM and cortex, respectively, and a is the stress-dependent elongation rate. Normalized Maximum Principal Stress is defined as the ratio of the maximum principal stress to the shear modulus of the ECM (μ_s). $T = G^{\mathrm{ctx}}t$ is dimensionless simulation time that ranges from 0 to 1, where G^{ctx} and t are cortex growth rate and time, respectively. As shown, increasing the cortex layer thickness leads to a longer folding wavelength and the emergence of larger folds. Additionally, the maximum principal stress increases slightly with the thicker cortex layer.

Table S3. Fiber density within gyri and sulci for ten random brains. Source data are provided as a Source Data file.

Case number	Fiber Density within Gyri (%)		Fiber Density within sulci (%)	
	Left	Right	Left	Right
#877168	82.65	64.57	17.35	35.43
#917255	78.25	87.20	21.75	12.80
#861456	85.73	75.01	14.27	24.99
#859671	89.11	86.91	10.89	13.09
#103818	80.39	71.00	19.61	19.00
#111312	83.69	73.65	16.31	26.35
#115320	79.93	74.80	20.07	25.20
#125525	86.08	79.59	13.92	20.41
#137128	88.85	78.18	11.15	21.82
#144226	87.34	68.09	12.66	31.91
Average	80.05		19.95	

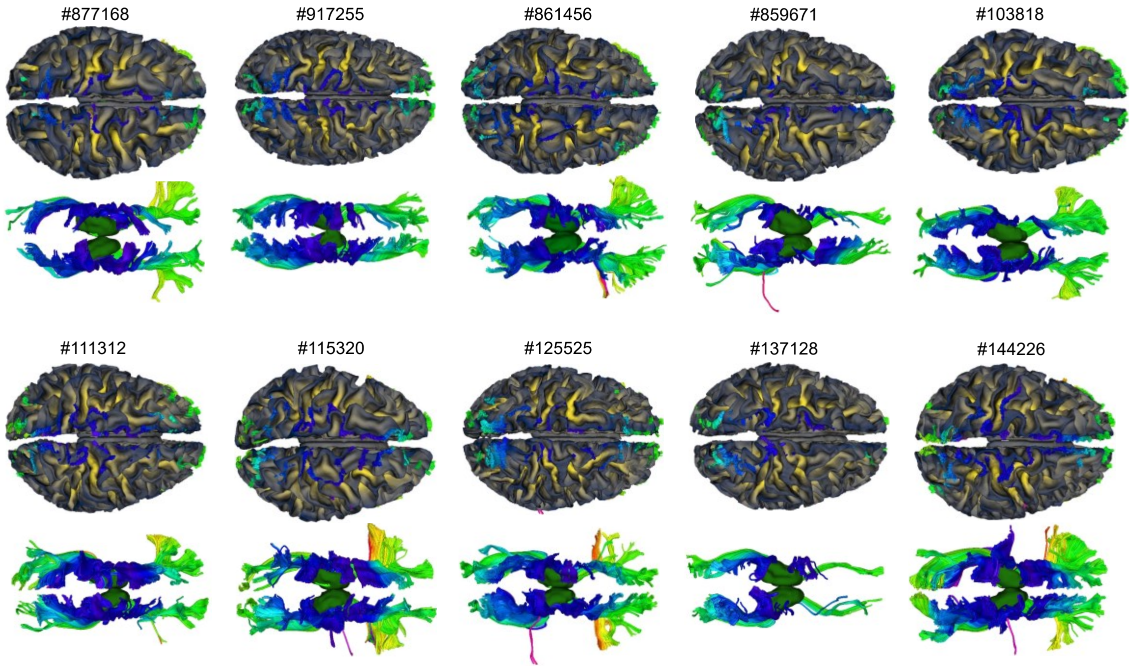

Fig. S4. Projection and distribution of thalamocortical fiber tracts on the white matter surface for 10 brains. Blue areas on the surfaces of the white matter indicate the tips of thalamocortical fibers. The MRI and diffusion MRI images shown in this figure were obtained from the publicly available Q1 release of the WU-Minn Human Connectome Project (HCP) database (https://humanconnectome.org/).

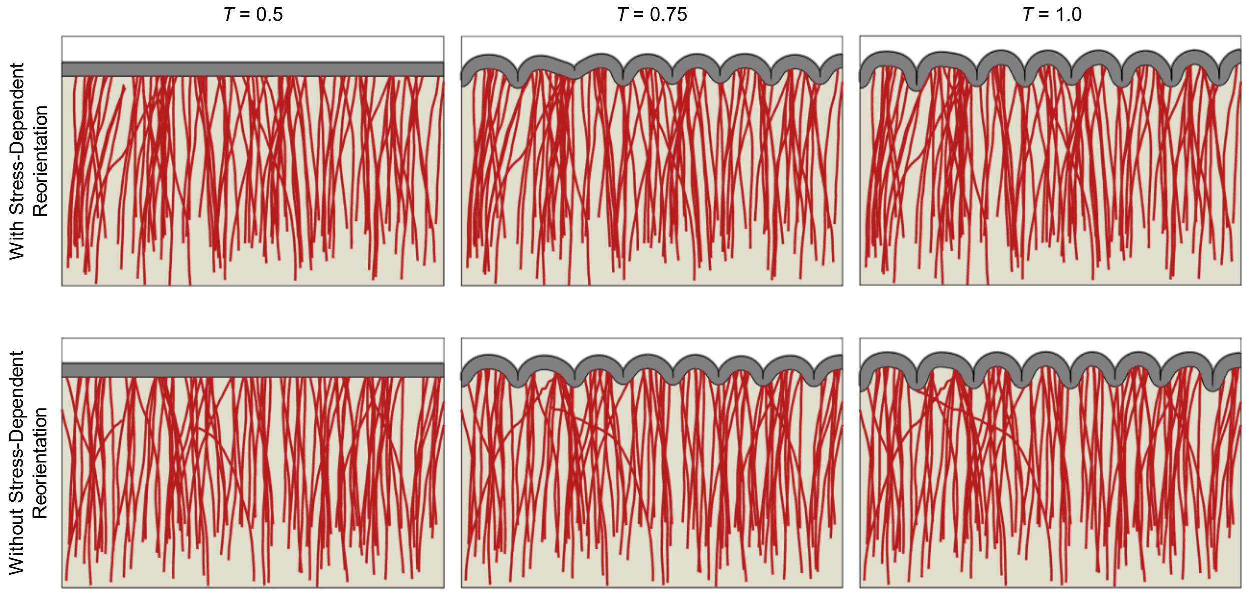

Fig. S5 Dynamic growth of fibers for the growth rate of $1.2\ \mathrm{mm\ d^{-1}}$ in models with (top) and without stress-dependent reorientation process (bottom). The used parameters in these figures are: $\mu_{\mathrm{f}}/\mu_{\mathrm{s}} = 2$, $\mu_c/\mu_s = 2$, $a = 0.015\ \mathrm{mm\ Pa^{-1}\ d^{-1}}$, $G^{\mathrm{axn}} = 1.2\ \mathrm{mm\ d^{-1}}$, where μ_{f}, μ_{s}, and μ_{c} are the shear moduli of the fiber, ECM,

and cortex, respectively, G^{axn} is the axon growth rate, and a is the stress-dependent elongation rate. $T = G^{\mathrm{ctx}} t$ is dimensionless simulation time that ranges from 0 to 1, where G^{ctx} and t are cortex growth rate and time, respectively.

Table S4. Algorithm update scheme for the reorientation of axon bundle

Internal variable $\mathbf{n}_k^{\mathrm{A}} = \left[n_{k1}^{\mathrm{A}},\ n_{k2}^{\mathrm{A}},\ n_{k3}^{\mathrm{A}}\right]^t$

1. Set initial values

   $\mathbf{n}^{\mathrm{A}} = \mathbf{n}_k^{\mathrm{A}}, \qquad \mathbf{T} = J^{\mathrm{e}-1}\mathbf{F}^{\mathrm{e}}.\frac{\partial W}{\partial \mathbf{F}^{\mathrm{eT}}}$

2. Compute principal stresses and directions

   $\mathbf{T} = J^{\mathrm{e}-1}\mathbf{F}^{\mathrm{e}}.\frac{\partial W}{\partial \mathbf{F}^{\mathrm{eT}}} = \sum_{i=1}^{3} \sigma_i \mathbf{n}_i \otimes \mathbf{n}_i$ with $\sigma_1 \geq \sigma_2 \geq \sigma_3$

   Let $\mathbf{n}_{\max}^T = \mathbf{n}_1^T$

   If $\sigma_1 < 0$ or $\sigma_1 = \sigma_2$ then

   $\dot{\mathbf{n}}^{\mathrm{A}} = 0$

   Else if $\mathbf{n}^{\mathrm{A}} \cdot \mathbf{n}_1^T < 0$ then

   $\mathbf{n}_{\max}^T \leftarrow -\mathbf{n}_1^T$

   Else

   $\mathbf{n}_{\max}^T$ remains as $\mathbf{n}_1^T$

   End if

3. Compute the magnitude and normal vector of rotation velocity

   $$\omega = \frac{\pi}{2t^*} \|\mathbf{n}^{\mathrm{A}} \times \mathbf{n}_{\max}^T\|$$

   $$\mathbf{n}^{\omega} = \frac{\mathbf{n}^{\mathrm{A}} \times \mathbf{n}_{\max}^T}{\|\mathbf{n}^{\mathrm{A}} \times \mathbf{n}_{\max}^T\|}$$

4. Update preferred direction vector

   $$\mathbf{n}_{k+1}^{\mathrm{A}} = \cos(\Delta t\omega)\,\mathbf{n}^{\mathrm{A}} + \sin(\Delta t\omega)\,\mathbf{n}^{\omega} \times \mathbf{n}^{\mathrm{A}} + [1 - \cos(\Delta t\omega)][\mathbf{n}^{\omega} \cdot \mathbf{n}^{\mathrm{A}}]\mathbf{n}^{\omega}$$

   $$\mathbf{n}_k^{\mathrm{A}} = \mathbf{n}_{k+1}^{\mathrm{A}}$$

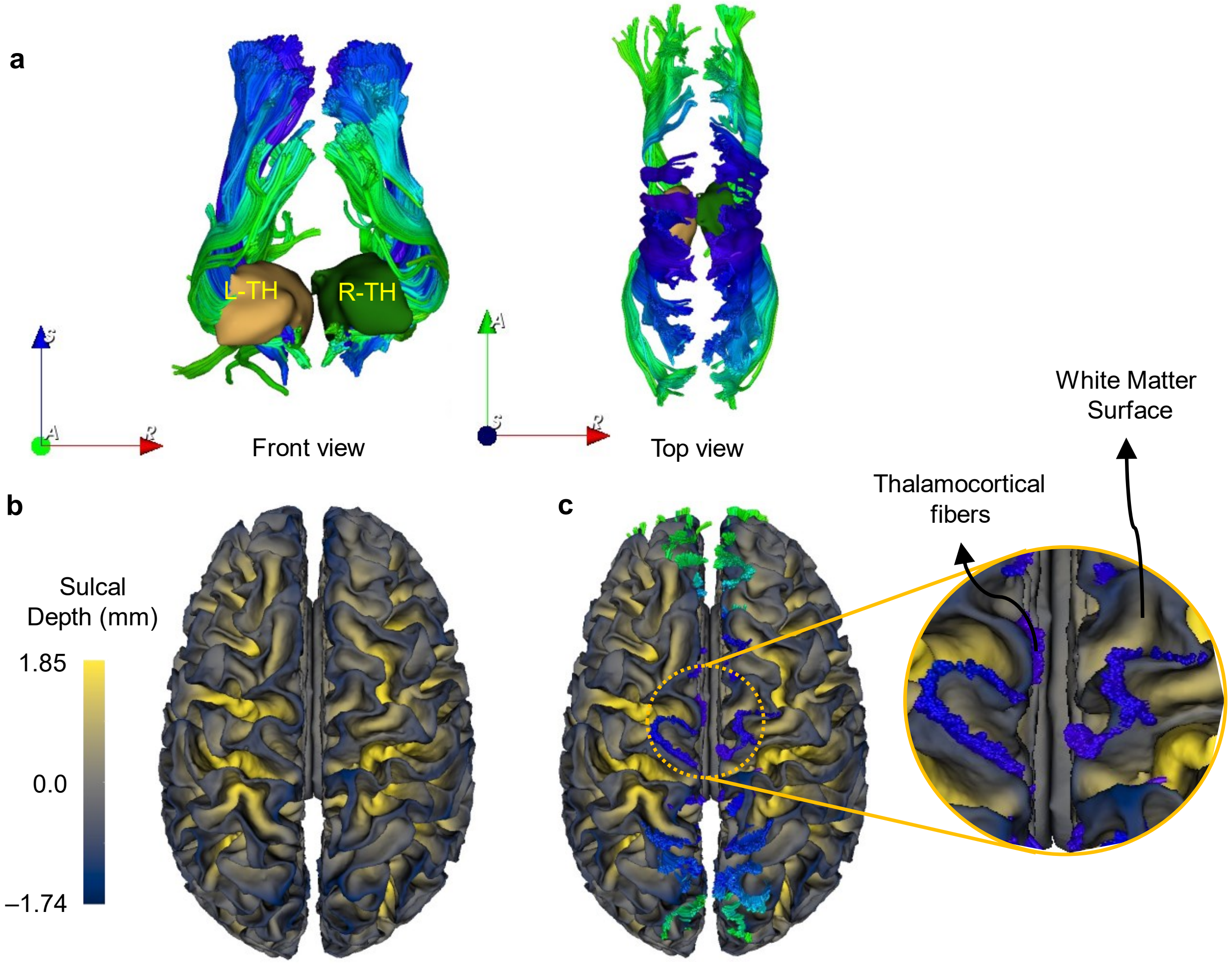

Fig. S6 Extraction of thalamocortical fiber tracts map. a Front and top views of the thalamocortical fiber tracts of a random brain case. b White matter surface of the brain in colors. The sulci depth was mapped to the surface. Dark blue regions represent the gyri and yellow regions show the sulci. c Projection and distribution of thalamocortical fiber tracts on the white matter surface for a brain sample. Blue areas in the zoomed-in image show that the thalamocortical fiber tracts follow gyral patterns. For better clarity, readers are referred to the color version of the figure in the online version of this article. The MRI and diffusion MRI images shown in this figure were obtained from the publicly available Q1 release of the WU-Minn Human Connectome Project (HCP) database (https://humanconnectome.org/).

References

1. Krsnik, Ž., Majić, V., Vasung, L., Huang, H. & Kostović, I. Growth of Thalamocortical Fibers to the Somatosensory Cortex in the Human Fetal Brain. *Front. Neurosci.* **11**, 233 (2017).
