# Supplementary material for: Stress landscape of folding brain serves as a map for axonal pathfinding
Source: Nat Commun. 2025 Jan 30;16:1187. doi: 10.1038/s41467-025-56362-3 (PMC11782574; doi:10.1038/s41467-025-56362-3)
Supplement: Supplementary file 2 — Description of Additional Supplementary Files [file 41467_2025_56362_MOESM2_ESM.pdf]

Description of Additional Supplementary Files

File name: Supplementary Movie 1
Description: Fiber growth and pathfinding within the stress landscape of the folding system.

File name: Supplementary Movie 2
Description: Dynamic fiber growth and pathfinding at a rate of 0.6 mm/day.

File name: Supplementary Movie 3
Description: Dynamic fiber growth and pathfinding at a rate of 1.2 mm/day.

File name: Supplementary Movie 4
Description: Dynamic fiber growth and pathfinding at a cortex-to-ECM stiffness ratio of 1.

File name: Supplementary Movie 5
Description: Dynamic fiber growth and pathfinding at a cortex-to-ECM stiffness ratio of 4.
