# Supplementary material for: Stress landscape of folding brain serves as a map for axonal pathfinding
Source: Nat Commun. 2025 Jan 30;16:1187. doi: 10.1038/s41467-025-56362-3 (PMC11782574; doi:10.1038/s41467-025-56362-3)
Supplement: Supplementary file 8 — Reporting Summary [file 41467_2025_56362_MOESM8_ESM.pdf]

nature portfolio

Corresponding author(s): Mir Jalil Razavi

Last updated by author(s): Dec 23, 2024

Reporting Summary

Nature Portfolio wishes to improve the reproducibility of the work that we publish. This form provides structure for consistency and transparency in reporting. For further information on Nature Portfolio policies, see our Editorial Policies and the Editorial Policy Checklist.

Statistics

For all statistical analyses, confirm that the following items are present in the figure legend, table legend, main text, or Methods section.

n/a	Confirmed	
☐	☒	The exact sample size (*n*) for each experimental group/condition, given as a discrete number and unit of measurement
☐	☒	A statement on whether measurements were taken from distinct samples or whether the same sample was measured repeatedly
☐	☒	The statistical test(s) used AND whether they are one- or two-sided<br>*Only common tests should be described solely by name; describe more complex techniques in the Methods section.*
☒	☐	A description of all covariates tested
☒	☐	A description of any assumptions or corrections, such as tests of normality and adjustment for multiple comparisons
☐	☒	A full description of the statistical parameters including central tendency (e.g. means) or other basic estimates (e.g. regression coefficient) AND variation (e.g. standard deviation) or associated estimates of uncertainty (e.g. confidence intervals)
☐	☒	For null hypothesis testing, the test statistic (e.g. *F*, *t*, *r*) with confidence intervals, effect sizes, degrees of freedom and *P* value noted<br>*Give P values as exact values whenever suitable.*
☒	☐	For Bayesian analysis, information on the choice of priors and Markov chain Monte Carlo settings
☒	☐	For hierarchical and complex designs, identification of the appropriate level for tests and full reporting of outcomes
☒	☐	Estimates of effect sizes (e.g. Cohen's *d*, Pearson's *r*), indicating how they were calculated

Our web collection on statistics for biologists contains articles on many of the points above.

Software and code

Policy information about availability of computer code

Data collection	Abaqus 2019, Custom user defined material VUMAT subroutine code, Slicer 5.6.1, ParaView 5.12.0-RC1
Data analysis	Matlab R2021a, IBM SPSS Statistics V 28.0.1.0 (001)

For manuscripts utilizing custom algorithms or software that are central to the research but not yet described in published literature, software must be made available to editors and reviewers. We strongly encourage code deposition in a community repository (e.g. GitHub). See the Nature Portfolio guidelines for submitting code & software for further information.

Data

Policy information about availability of data

All manuscripts must include a data availability statement. This statement should provide the following information, where applicable:

- Accession codes, unique identifiers, or web links for publicly available datasets
- A description of any restrictions on data availability
- For clinical datasets or third party data, please ensure that the statement adheres to our policy

https://github.com/akbarsolhtalab/Axonal-PathFinding

Research involving human participants, their data, or biological material

Policy information about studies with human participants or human data. See also policy information about sex, gender (identity/presentation), and sexual orientation and race, ethnicity and racism.

Reporting on sex and gender	In this study, structural MRI (sMRI) and diffusion MRI (dMRI) scans were obtained from the WU-Minn Human Connectome Project (HCP). The sample consists of 10 healthy adult brains. The HCP database includes detailed information about sex and gender; however, specific sex and gender data for the selected sample were not the focus of this study.
Reporting on race, ethnicity, or other socially relevant groupings	The WU-Minn Human Connectome Project (HCP) database includes diverse participants. For this study, the specific race, ethnicity, or other socially relevant groupings of the 10 healthy adult brains aged 22–35 years were not the focus and were not explicitly considered in the analysis.
Population characteristics	The sample consisted of 10 healthy adult brains, aged 22–35 years. These individuals were part of the Q1 release of the WU-Minn Human Connectome Project (HCP), which is known for its high-quality imaging data and thorough demographic documentation.
Recruitment	Participants were recruited as part of the WU-Minn Human Connectome Project (HCP)
Ethics oversight	Washington University in St. Louis

Note that full information on the approval of the study protocol must also be provided in the manuscript.

Field-specific reporting

Please select the one below that is the best fit for your research. If you are not sure, read the appropriate sections before making your selection.

☒ Life sciences ☐ Behavioural & social sciences ☐ Ecological, evolutionary & environmental sciences

For a reference copy of the document with all sections, see nature.com/documents/nr-reporting-summary-flat.pdf

Life sciences study design

All studies must disclose on these points even when the disclosure is negative.

Sample size	A total of 10 healthy adult brains aged 22–35 years were included in this study. The sample size was determined based on the availability of high-quality imaging data from the Q1 release of the WU-Minn Human Connectome Project (HCP). This size was deemed sufficient for the initial analysis conducted.
Data exclusions	No data were excluded from the analyses. All 10 sets of MRI and DTI images collected from the HCP database were used in the study.
Replication	This study utilized existing data from the WU-Minn Human Connectome Project (HCP). The replication of findings is feasible using the same dataset, which is publicly available for other researchers to access and verify results. No experimental replication was performed as this study is based on secondary data analysis.
Randomization	We randomly selected 10 healthy adult brain from the HCP database.
Blinding	Blinding was not applicable in this study as it involved the analysis of pre-existing imaging data. The researchers did not have any influence over data collection or participant selection, as these were conducted by the HCP team.

Reporting for specific materials, systems and methods

We require information from authors about some types of materials, experimental systems and methods used in many studies. Here, indicate whether each material, system or method listed is relevant to your study. If you are not sure if a list item applies to your research, read the appropriate section before selecting a response.

Materials & experimental systems

n/a	Involved in the study	
☒	☐	Antibodies
☒	☐	Eukaryotic cell lines
☒	☐	Palaeontology and archaeology
☒	☐	Animals and other organisms
☒	☐	Clinical data
☒	☐	Dual use research of concern
☒	☐	Plants

Methods

n/a	Involved in the study	
☒	☐	ChIP-seq
☒	☐	Flow cytometry
☐	☒	MRI-based neuroimaging

Plants

Seed stocks

Report on the source of all seed stocks or other plant material used. If applicable, state the seed stock centre and catalogue number. If plant specimens were collected from the field, describe the collection location, date and sampling procedures.

Novel plant genotypes

Describe the methods by which all novel plant genotypes were produced. This includes those generated by transgenic approaches, gene editing, chemical/radiation-based mutagenesis and hybridization. For transgenic lines, describe the transformation method, the number of independent lines analyzed and the generation upon which experiments were performed. For gene-edited lines, describe the editor used, the endogenous sequence targeted for editing, the targeting guide RNA sequence (if applicable) and how the editor was applied.

Authentication

Describe any authentication procedures for each seed stock used or novel genotype generated. Describe any experiments used to assess the effect of a mutation and, where applicable, how potential secondary effects (e.g. second site T-DNA insertions, mosiacism, off-target gene editing) were examined.

Magnetic resonance imaging

Experimental design

Design type

Indicate task or resting state; event-related or block design.

Design specifications

This study analyzed pre-existing structural MRI (sMRI) and diffusion MRI (dMRI) data obtained from the WU-Minn Human Connectome Project (HCP) to investigate brain structure and connectivity in healthy adults.

Behavioral performance measures

Behavioral performance measures were not the focus of this study. The study exclusively analyzed neuroimaging data to understand brain structure and connectivity.

Acquisition

Imaging type(s)

Structural MRI (sMRI) and diffusion MRI (dMRI)

Field strength

3 Tesla (3T)

Sequence & imaging parameters

For structural MRI (sMRI):
T1-weighted imaging:
Repetition Time (TR): 2400 ms
Flip Angle: 8 degrees
Voxel Size: 0.7 mm isotropic

Area of acquisition

The HCP team has scanned the entire brain for both sMRI and dMRI.

Diffusion MRI ☒ Used ☐ Not used

Parameters

For diffusion MRI (dMRI):
Repetition Time (TR): 5520 ms
Voxel Size: 1.25 mm isotropic
b-values: 1000, 2000, 3000 s/mm²
Number of diffusion directions: 90

Preprocessing

Preprocessing software

FreeSurfer

Normalization

If data were normalized/standardized, describe the approach(es): specify linear or non-linear and define image types used for transformation OR indicate that data were not normalized and explain rationale for lack of normalization.

Normalization template

Describe the template used for normalization/transformation, specifying subject space or group standardized space (e.g. original Talairach, MNI305, ICBM152) OR indicate that the data were not normalized.

Noise and artifact removal

Describe your procedure(s) for artifact and structured noise removal, specifying motion parameters, tissue signals and physiological signals (heart rate, respiration).

Volume censoring

Define your software and/or method and criteria for volume censoring, and state the extent of such censoring.

Statistical modeling & inference

Model type and settings

A thorough statistical analysis of the study's results was performed using SPSS to explore how the biophysical properties of axons, cortex, and white matter extracellular matrix (ECM) impact cortical folding and fiber organization. Tukey's Honestly Significant Difference (HSD) test was utilized with a significance level of 0.06 and 94% confidence intervals. This approach was

employed to compare means across multiple groups, making it especially useful for detecting statistically significant differences between pairs of groups.

Effect(s) tested

Define precise effect in terms of the task or stimulus conditions instead of psychological concepts and indicate whether ANOVA or factorial designs were used.

Specify type of analysis: ☐ Whole brain ☒ ROI-based ☐ Both

Anatomical location(s): In this study, we have focused on Thalamacortical fibers.

Statistic type for inference
(See Eklund et al. 2016)

Specify voxel-wise or cluster-wise and report all relevant parameters for cluster-wise methods.

Correction

Describe the type of correction and how it is obtained for multiple comparisons (e.g. FWE, FDR, permutation or Monte Carlo).

Models & analysis

n/a	Involved in the study
☒	☐ Functional and/or effective connectivity
☒	☐ Graph analysis
☒	☐ Multivariate modeling or predictive analysis
